# Burden of Disease from Toxic Waste Sites in India, Indonesia, and the Philippines in 2010

**DOI:** 10.1289/ehp.1206127

**Published:** 2013-05-04

**Authors:** Kevin Chatham-Stephens, Jack Caravanos, Bret Ericson, Jennifer Sunga-Amparo, Budi Susilorini, Promila Sharma, Philip J. Landrigan, Richard Fuller

**Affiliations:** 1 Department of Preventive Medicine, Icahn School of Medicine at Mount Sinai, New York, New York, USA; 2 School of Public Health at Hunter College, City University of New York, New York, New York, USA; 3 Blacksmith Institute, New York, New York, USA; 4 Department of Social Development Services, University of the Philippines Los Baños, Los Baños, Philippines; 5 Department of Pediatrics, Icahn School of Medicine at Mount Sinai, New York, New York, USA

**Keywords:** Asia, burden of disease, chemical exposure, disability-adjusted life year, toxic waste sites

## Abstract

Background: Prior calculations of the burden of disease from toxic exposures have not included estimates of the burden from toxic waste sites due to the absence of exposure data.

Objective: We developed a disability-adjusted life year (DALY)-based estimate of the disease burden attributable to toxic waste sites. We focused on three low- and middle-income countries (LMICs): India, Indonesia, and the Philippines.

Methods: Sites were identified through the Blacksmith Institute’s Toxic Sites Identification Program, a global effort to identify waste sites in LMICs. At least one of eight toxic chemicals was sampled in environmental media at each site, and the population at risk estimated. By combining estimates of disease incidence from these exposures with population data, we calculated the DALYs attributable to exposures at each site.

Results: We estimated that in 2010, 8,629,750 persons were at risk of exposure to industrial pollutants at 373 toxic waste sites in the three countries, and that these exposures resulted in 828,722 DALYs, with a range of 814,934–1,557,121 DALYs, depending on the weighting factor used. This disease burden is comparable to estimated burdens for outdoor air pollution (1,448,612 DALYs) and malaria (725,000 DALYs) in these countries. Lead and hexavalent chromium collectively accounted for 99.2% of the total DALYs for the chemicals evaluated.

Conclusions: Toxic waste sites are responsible for a significant burden of disease in LMICs. Although some factors, such as unidentified and unscreened sites, may cause our estimate to be an underestimate of the actual burden of disease, other factors, such as extrapolation of environmental sampling to the entire exposed population, may result in an overestimate of the burden of disease attributable to these sites. Toxic waste sites are a major, and heretofore underrecognized, global health problem.

Toxic waste sites threaten the environment and human health in countries around the world. In developing countries these sites—and their risks to human health—have not been optimally assessed (Yáñez et al. 2002). Quantification of the burden of disease from toxic waste sites can assist public health planning and remediation efforts by complementing traditional waste site investigations and by framing these toxic exposures in the context of other exposures. Burden of disease estimates are typically expressed in disability-adjusted life years (DALYs). The DALY metric accounts for both the morbidity and mortality that result from a disease, injury, or health state (Prüss-Üstün et al. 2003).

Previous calculations of the burden of disease from toxic exposures have not included estimates from toxic waste sites because of an absence of data on exposures and health impacts. In 2004, Fewtrell et al. (2004) estimated that lead causes nearly 1% of the global burden of disease. Then in 2011, Prüss-Üstün et al. (2011) calculated that exposure to a variety of chemicals, including lead, secondhand smoke, and asbestos, accounts for 5.7% of total global DALYs and 8.3% of total global deaths. However, because of insufficient data, neither of these studies included estimates for disease and death attributable to exposures from toxic waste sites.

We aimed to develop a DALY-based estimate of the burden of disease and death attributable to toxic waste sites in India, Indonesia, and the Philippines. To our knowledge, no systematic evaluation of toxic waste sites in low- and middle-income countries (LMICs) had previously been performed. The paucity of data has precluded calculation of the burden of disease resulting from exposures at these sites. Through this effort we hope to ultimately calculate the contribution of toxic waste sites to the global burden of disease.

## Methods

*Site identification*. In this study, we utilized data collected through Blacksmith Institute’s Toxic Sites Identification Program (TSIP), an effort to identify and screen contaminated sites in LMICs (Blacksmith Institute 2013). The TSIP, which is implemented jointly with the United Nations Industrial Development Organization, identifies point-source pollution from industrial sites that present a public health risk. A particular focus is placed on abandoned (legacy) sites, such as former tanneries, as well as small-scale artisanal sources, such as lead battery recycling and artisanal gold mining. Although other sources of contamination, such as large-scale mining, may also be included and screened, the majority of sites come from these two categories (i.e., legacy sites and artisanal sources). The TSIP excludes nonpoint sources, such as ambient urban air pollution, and non-chemical contamination, such as sewage-contaminated water. Ericson et al. (2012) described the types of sites identified in the TSIP.

The Blacksmith Institute developed an evaluation instrument, the Initial Site Screening (ISS), for rapid data collection and assessment of these sites (Blacksmith Institute 2013). The ISS is a modified and simplified version of the U.S. Environmental Protection Agency’s (EPA) Hazard Ranking System, used to prioritize and rank toxic waste sites in the U.S. EPA’s Superfund program (U.S. EPA 2012b). The ISS includes information on the concentration of the key toxic chemical, the primary environmental medium of the exposure pathway, and the size of the population at risk.

To undertake the TSIP, the Blacksmith Institute contracted and trained approximately 150 site investigators. These investigators identified and visited sites, collected environmental samples, took photographs and GPS coordinates, interviewed stakeholders, and categorized the potential contaminated environmental media. After being educated on the project and assured that participation in the interview process was voluntary, the stakeholders agreed to participate; written informed consent was not obtained. The investigators determined the dominant pollutant for each site based in part on prior testing or historical use of a site, then took samples to measure levels of the pollutant, typically in only one environmental medium. For sites where only total chromium was reported, the speciation coefficient of 0.6 was used to estimate hexavalent chromium (Avudainayagam et al. 2003; Kumar and Riyazuddin 2010). Between 2009 and 2012, investigators completed 1,510 such screenings in 49 countries. Since the majority of screenings occurred in 2010, we used 2010 as our baseline year for analysis. We have previously described the ISS protocol and TSIP in detail (Ericson et al. 2012).

*Population at risk of exposure*. As part of the ISS, investigators estimated the population at risk of exposure for each site, indicating the number of persons regularly coming into contact with the contaminant in the relevant environmental medium. For example, if water contamination is documented, then the population at risk includes those individuals who use the water daily for drinking, food preparation, and other domestic purposes. Investigators used a range of approaches to obtain this information, including visual methods, satellite photographs, community census data, government interviews, and personal knowledge. The age distribution at sites was not recorded as part of the ISS. Therefore, we applied age distribution estimates from the U.S. Census Bureau (2012) for each country to the population around each site within that country. We divided each site’s estimated population into 17 age groups based on these distributions (e.g., 0–4, 5–9, 10–14 years).The World Health Organization (WHO) DALY calculator for cardiovascular disease resulting from adult lead exposure uses 5 age groups (i.e., 15–29, 30–44, 45–59, 60–69, 70–79 years). In this study, we condensed the 17 age groups into the appropriate 5 groups to enable our calculations.

*Calculating risk per person*. We divided human health effects into cancer and noncancer effects. For carcinogens, we used the U.S. EPA’s Regional Screening Level Calculator for Chemical Contaminants to calculate long-term cancer risk per unit toxicant (i.e., cancer probability per milligram per kilogram soil for agents found in soil or microgram per liter water for waterborne agents) (U.S. EPA 2012c). For noncancer health effects, reference doses (RfDs) and concentrations (RfCs) from the U.S. EPA’s Integrated Risk Information System (IRIS) database were applied to the exposure pathways and contamination levels at each site (U.S. EPA 2012a). The modeling assumed a linear dose response and used the health outcome associated with the RfD or RfC (e.g., liver toxicity, renal toxicity). A listing of the cancer and noncancer risks per unit of contaminant, with the exception of lead, is presented in Table 1. Given the availability of lead-specific modeling tools and dose–response relationships, we calculated disease incidence and DALYs from lead separately.

**Table 1:** Per capita cancer and noncancer human health risks by chemical and media for chemicals other than lead.

Chemical (media assessed)	Cancer risk			Noncancer risk		
	Per µg/m^3^ in air	Permg/kg in soil	Per µg/L inwater	Per µg/m^3^ in air	Permg/kg in soil	Per µg/L inwater
Aldrin (W)	NA	NA	5.35 × 10^–4^	NA	NA	2.22 × 10^–6^
Asbestos (A)	2.30 × 10^–1^^*a*^	NA	NA	NA	NA	NA
Cadmium (A,S,W)	1.80 × 10^–3^	NA	NA	5.00 × 10^–5^	2.67 × 10^–8^	1.33 × 10^–7^
Chromium VI (A,S,W)	8.40 × 10^–2^	9.71 × 10^–8^^*b*^	2.09 × 10^–5^	NA	NA	NA
DDT (W)	NA	NA	1.07 × 10^–5^	NA	NA	1.33 × 10^–7^
Lindane (S,W)	NA	5.08 × 10^–6^	3.45 × 10^–5^	NA	8.85 × 10^–8^	2.22 × 10^–7^
Mercury, inorganic (A,S,W)	NA	NA	NA	5.68 × 10^–8^	8.85 × 10^–8^	2.22 × 10^–7^
Abbreviations: A, air; DDT, dichlorodiphenyltrichloroethane; NA, not assessed; S, soil; W, water.^***a***^Fibers/cubic centimeter. ^***b***^Inhaled airborne dust.						

*Calculating incidence of disease*. For each chemical, we considered up to three environmental media (soil, water, air) and corresponding routes of exposure (ingestion, dermal, and/or inhalation). To calculate disease incidence for all chemicals except lead, we multiplied the risk per person by the level of the contaminant in the relevant environmental medium. Because linear slope factors were utilized to calculate incidence, very high concentrations of contaminants resulted in correspondingly high estimates of disease incidence. To accommodate this limitation of the model, we arbitrarily capped incidence for all diseases at 5%.

For lead, we calculated the incidence of mild mental retardation and anemia in children and cardiovascular disease in adults resulting from lead-induced increases in blood pressure. We calculated the predicted mean blood lead levels (BLLs) that would result from lead exposures at each site by entering the soil and drinking-water lead levels measured at each site into the U.S. EPA’s Integrated Exposure, Uptake and Biokinetic (IEUBK) model for lead and Adult Lead Methodology (ALM) (Caravanos et al. 2012; U.S. EPA 1994; White et al. 1998). We calibrated default soil ingestion levels in the IEUBK model upward from 200 mg/day to 400 mg/day. This approach follows similar analyses done in Native American populations (400 mg/day), as well as in indigenous populations in Micronesia (500 mg/day), and is above the “upper bound” level (200 mg/day) used by the U.S. EPA (Harris and Harper 2004; Sun and Meinhold 1997; U.S. EPA 2011). Then we calculated the incidence of mild mental retardation and cardiovascular outcomes that would result from such BLLs, using spreadsheets developed by the WHO (2013). We also assumed that 20% of children with BLLs > 70 µg/dL develop anemia (Fewtrell et al. 2003).

*Calculating years lived with disability (YLDs) and years of life lost (YLLs)*. The DALY metric is the sum of two components: YLD, which represents disease-related morbidity, and YLL, which represents the premature mortality from the disease. We calculated YLD and YLL for exposure to each contaminant through each relevant environmental medium. YLD is the product of the estimated years lived with a given disability multiplied by its specific disability weight (DW). The DW is a value from zero to one, depending on the severity of each disease, with zero representing ideal health and one representing death. For example, periodontal disease has a DW of 0.001, whereas a first-time stroke has a DW of 0.920 (WHO 2008).

For each chemical, we assigned the relevant type of cancer, noncancer health effect, and corresponding DW (Table 2) (U.S. EPA 2012a; WHO 2008). If the chemical’s health effect did not align with a disease in the WHO DW database, then we selected the most appropriate disease and DW on the basis of the target organ, duration of disease, and severity of disease. In the case of noncarcinogenic effects, the total number of years of life remaining at onset was multiplied by the appropriate DW to determine YLD (Prüss-Üstün et al. 2003). We chose to apply the exposure for the remainder of an individual’s life expectancy given that most LMICs do not have a systemic program to identify and remediate these sites. For carcinogens, we applied a DW and duration to each cancer stage: diagnosis (cancer-specific DW; 3 years); metastasis (DW 0.75; 1 year); and terminal (DW 0.81; 1 year) (WHO 2008). YLLs were calculated only for carcinogens. We used cancer incidence and survival data to calculate the resulting number of deaths (Ferlay et al. 2010; Sankaranarayanan et al. 2010). All cancers were assumed to last 5 years, before either going into remission or resulting in death.

**Table 2:** Cancer and noncancer health effects and DWs of chemicals found at waste sites.

Chemical	Cancer site (classification)^*a*^	Cancer-specific DW^*b*^	Health effect (noncancer)	DW (noncancer)
Aldrin (W)	Liver (probable)	0.20	Liver toxicity	0.104^*c*^
Asbestos (A)	Lung (confirmed)	0.15	NA	NA
Cadmium (A)	Lung (probable)	0.15	NA	NA
Cadmium (W,S)	NA	NA	Renal toxicity	0.091^*d*^
Chromium VI (A,W,S)	Lung (confirmed)	0.15	NA	NA
DDT (W)	Liver (probable)	0.20	Liver toxicity	0.104^*c*^
Lead (A,W,S)	NA	NA	Mild mental retardation	0.361
Decrement in IQ	0.024^*e*^
Cardiovascular disease	NA^*f*^
Anemia	0.024
Lindane (W,S)	Liver (possible)	0.20	Liver toxicity	0.104^*c*^
Mercury, inorganic (W,S)	NA	NA	Renal toxicity	0.091^*d*^
Abbreviations: A, air; DDT, dichlorodiphenyltrichloroethane; DW, disability weight; NA, not assessed; S, soil; W, water.^***a***^Human carcinogenicity classification (U.S. EPA 2012a). ^***b***^Cancer-specific DW was applied for a duration of 3years, then a DW of 0.75 was applied for 1year (metastasis), followed by a DW of 0.81 for 1year (terminal stage). ^***c***^Advanced hepatic disease. ^***d***^Acute glomerulonephritis. ^***e***^Developmental disability associated with protein–energy malnutrition. ^***f***^DALYs calculated with the environmental attributable fraction approach.				

For lead, we utilized the environmentally attributable fraction approach in determining the contribution of lead exposure to the burden of cardiovascular disease (ischemic heart disease, cerebrovascular disease, hypertensive disease, and other cardiac disease) (Fewtrell et al. 2003). The WHO has calculated the fraction of cardiovascular disease attributable to lead exposure based on BLL. By entering the predicted BLL and total cardiovascular disease DALYs for each country into a WHO spreadsheet, we calculated the DALYs attributable to cardiovascular disease from lead exposure at toxic waste sites in each of the three countries. In addition, for children with BLLs > 10 µg/dL who did not have mental retardation, we applied the DW for developmental disability from protein–energy malnutrition (0.024) as a proxy DW for lifelong disability from IQ loss in the absence of mental retardation. Prior research suggests that the loss of IQ points may impact cardiovascular and all-cause mortality, resulting in increased morbidity and mortality (Batty et al. 2010; Lager et al. 2009).

We then applied weighting factors to the resulting YLD and YLL for each chemical, including a discount rate to account for inherent inaccuracies when predicting future events, and age weights to reflect the relative societal value of different age groups (Mathers et al. 2006). The notation DALYs_(r,K)_ signifies the discount rate (r) and age weight (K) used. Our primary results are expressed as DALYs_(3,1)_, which include a 3% discount rate and the full age weight. We also calculated DALYs_(3,0)_ with the 3% discount rate only, and DALYs_(0,0)_ without any weighting to provide a range of estimates (Mathers et al. 2006).

For example, the drinking water at one site in India had an aldrin level of 0.063 ppb. The oral RfD for aldrin for liver toxicity is 3.0 × 10^–5^ mg/kg/day, which converts to a risk of 8.57 × 10^–10^ per microgram per liter of drinking water (U.S. EPA 2012a, 2012c). The DW for advanced hepatic disease is 0.104. Assuming the 4,000 persons potentially exposed consume 2 L of drinking water each day, we calculated 1.62 DALYs_(3,0)_, 1.64 DALYs_(3,1)_, and 2.02 DALYs_(0,0)_ resulting from exposure to aldrin in drinking water at this site.

*Sensitivity analysis*. In addition to calculating DALYs with varying rates and weights, we also altered inputs into our model to conduct a sensitivity analysis. We varied the total population at risk by 25%, changed the disease incidence cap from the default value of 5% to 2.5% or 7.5%, and removed the additional DW for lead-induced IQ (intelligence quotient) losses that did not result in mild mental retardation. For a remediation scenario, we also assumed that remediation had reduced all pollutants to concentrations below international standards (Blacksmith Institute 2011). By subtracting the resulting DALYs from our primary estimate, we quantified the potential impact of remediating these sites.

We also estimated that an additional 5,000 unscreened sites exist in these countries, and that these sites present similar conditions as the screened sites. The TSIP prioritized screenings in part by the scale of the problem, measured in population at risk. Thus, these 5,000 sites are unlikely to have comparably large populations. We therefore assumed that the population at risk for each of these additional sites was the median of the population at risk of screened sites, which is lower than the mean population for screened sites. By contrast, the DALY per person estimates for the 5,000 unscreened sites are unlikely to be lower than those identified at the screened sites. Sites were not prioritized for screening based on the level of the contaminant in the pathway. Therefore, we applied the average DALY per person for the screened sites to the population at the unscreened sites.

## Results

**Figure 1:**
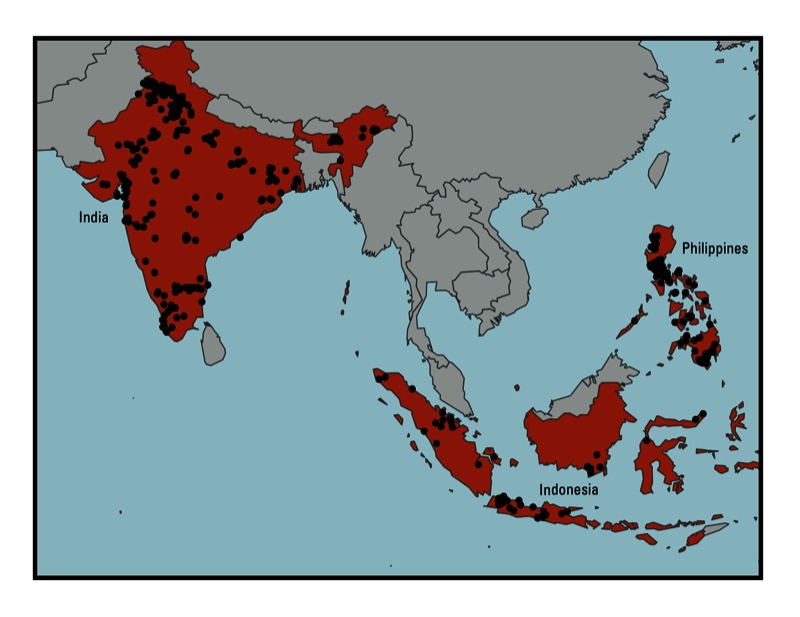
Locations of 373 toxic waste sites in India, Indonesia, and the Philippines in 2010.

*Sites evaluated*. Blacksmith Institute–trained investigators screened 498 sites in India, Indonesia, and the Philippines, with an estimated population at risk of exposure of approximately 12 million. Of the 23 separate chemicals documented at these sites, 8 occurred at more than one site and had established dose–response relationships correlating exposure with specific outcomes. We included in the analysis only the 373 sites containing 1 of these 8 chemicals. Figure 1 displays the geographical distribution of the sites in India (*n* = 221), Indonesia (*n* = 73), and the Philippines (*n* = 79). The estimated population at risk of exposure at these 373 sites was 8,629,750 (mean, 23,136, median, 7,000), which is 0.61% of the total population of the three countries. Of the exposed population, 3,449,592 were < 18 years of age and 2,184,220 were women of childbearing age (15–49 years of age). We estimated that an additional 5,000 unscreened sites exist in the three countries, with a population of 7,000 persons per site. This additional population equals 35,000,000, resulting in a total population of 43,629,750 for the screened and unscreened sites.

*YLD and YLL at screened sites*. We estimated 588,112 person-years lived with disease and 240,610 person-years lost as a result of chemical exposures in 2010 at the 373 toxic waste sites (Table 3). According to our estimates, lead was the largest contributor of the eight chemicals to YLD (523,630 YLD, 89% of total YLD), and hexavalent chromium was the largest contributor to YLL (235,483 YLL, 97.9% of total YLL). In Table 3, inhalation of soil and dust is incorporated into the soil results.

**Table 3:** YLDs, YLLs, and DALYs by chemical.

Chemical	No. of sites	Estimated population at risk	YLDs	YLLs	DALYs
Aldrin	5	133,000	212	812	1,024
Asbestos	3	25,000	974	4,218	5,192
Cadmium	53	976,600	15 (S=1, W=14)	0	15
Chromium VI	128	3,231,750	63,174 (S=3,582, W=59,592)	235,483 (S=14,467, W=221,016)	298,657
DDT	4	180,000	4	18	22
Lead	79	1,829,900	523,630	0	523,630
Lindane	9	131,300	20 (S=2, W=18)	79 (S=6, W=73)	99
Mercury, inorganic	92	2,122,200	83 (S=32, W=51)	0	83
Total	373	8,629,750	588,112	240,610	828,722
Abbreviations: DDT, dichlorodiphenyltrichloroethane; S, soil; W, water.					

*Premature deaths and DALYs at screened and unscreened sites*.We estimated that 828,722 DALYs_(3,1)_ resulted from chemical exposures at the 373 sites in 2010. By applying the value of 0.10 DALYs_(3,1)_ per person from the screened sites to the population at the unscreened sites, we estimated that 3,500,000 DALYs_(3,1)_ resulted from exposure at the unscreened sites. The total estimated DALYs_(3,1)_ for the screened and unscreened sites was 4,328,722. We also calculated that 66,747 persons would die prematurely from cancer, specifically liver and lung cancer, from exposures at these sites.

*Sensitivity analysis*. Removal of age weights yielded 814,934 DALYs_(3,0)_, whereas removal of age weights and the discount rate yielded 1,557,121 DALYs_(0,0)_ (Table 4). If the actual exposed population around these sites is 25% less or 25% greater than our estimate, the resulting DALYs_(3,1)_ would be 621,541 and 1,035,902, respectively. If the additional DW for lead-induced IQ loss not resulting in mental retardation is removed, our overall estimate would be 483,201 DALYs_(3,1)_. In addition, if disease incidence is capped at 2.5% or 7.5%, the resulting DALYs_(3,1)_ would be 730,627 and 922,479, respectively. The remediation scenario yielded 30,317 DALYs_(3,1)_, in contrast with our primary estimate of 828,722 DALYs_(3,1)_. Thus, our estimates suggest that 798,405 DALYs_(3,1)_ could be eliminated by remediation of these sites to achieve international standards.

**Table 4:** Sensitivity analysis estimates.

Scenario	Total DALYs
Primary estimate of screened sites	828,722 DALYs_(3,1)_
Estimate without age weights	814,934 DALYs_(3,0)_
Estimate without age weights or discount rate	1,557,121 DALYs_(0,0)_
Remediation scenario	30,317 DALYs_(3,1)_
If actual exposed population is 25% less	621,541 DALYs_(3,1)_
If actual exposed population is 25% greater	1,035,902 DALYs_(3,1)_
If additional DW for lead-induced IQ loss not resulting in MMR is removed	483,201 DALYs_(3,1)_
If incidence is capped at 2.5%	730,627 DALYs_(3,1)_
If incidence is capped at 7.5%	922,479 DALYs_(3,1)_
Estimate of unscreened sites	3,500,000 DALYs_(3,1)_
Estimate of screened and unscreened sites	4,328,722 DALYs_(3,1)_

## Discussion

We estimated that 8,629,750 persons were at risk of exposure to one of eight industrial pollutants at 373 toxic waste sites in three countries in 2010, resulting in 828,722 DALYs_(3,1)_. This estimate represents a burden of disease equal to 0.22% of the total estimated DALYs_(3,1)_ from all causes in India, Indonesia, and the Philippines (WHO 2008). Alteration of the discount rate and age weight leads to a range of estimates, from 814,934 DALYs_(3,0)_ to 828,722 DALYs_(3,1)_ to 1,557,121 DALYs_(0,0)_. Lead and hexavalent chromium account for 99.2% of the total DALYs estimated for the 8 waste site chemical exposures evaluated. The additional DW for lead-induced IQ loss not resulting in mental retardation accounts for 483,201 DALYs_(3,1),_ which represents approximately 58% of total DALYs_(3,1)_. Inclusion of an estimated number of unscreened sites increased the estimated population at risk of exposure to 43,629,750, and the total DALYs_(3,1)_ to 4,328,722. As part of a larger project attempting to calculate the burden of disease of toxic waste sites in LMICs, the present analysis indicates that the burden of disease associated with these sites is substantial and comparable to well-described diseases and environmental risk factors. For example, the WHO (2009) estimated that outdoor air pollution causes 1,448,612 DALYs_(3,1)_ and malaria causes 725,000 DALYs_(3,1)_ in these three countries. Overall, the present analysis begins to address the paucity of knowledge regarding health effects from toxic waste sites in LMICs and helps frame this issue in the context of other public health problems.

Given the limited scope of this project and the understanding that the screened sites represent only a portion of the total existing sites, we estimated that 5,000 unscreened sites exist in these three countries. The U.S. EPA (2004) estimates that there are approximately 294,000 contaminated sites in the United States alone that require some form of remediation. India’s population is nearly four times that of the United States, with nearly one third of Indian urban residents living in informal housing settlements, where unregulated cottage industries can proliferate without zoning or emissions controls (UN-HABITAT 2007).

Pollutants at toxic waste sites in LMICs can potentially have profound health effects. Lead and cadmium adversely affect neurodevelopment in children, with the *in utero* period being the life stage of greatest vulnerability (Ciesielski et al. 2012; Hu et al. 2006). Children and women of childbearing age constitute 65.3% of the total exposed population in this analysis, highlighting the potential impact on these vulnerable populations. The majority of the chemicals are nephrotoxic or hepatotoxic, and kidney and liver toxicity accounted for the majority of noncancer health effects. Several are known carcinogens, including asbestos, cadmium, and chromium.

Previous work has described the difficulty in identifying which toxic chemicals are being generated in India via industrial processes, as well as which ones are being imported for recycling or disposal (Dutta et al. 2006). The actual amount being produced and imported, and the ultimate fate of many of these chemicals, is unclear. Misra and Pandey (2005) discussed the complex requirements for proper handling of toxic waste to prevent human exposures and highlight the barriers to achieving this goal in countries such as India. Waste is often handled without adequate control mechanisms, such as proper infrastructure and personal protective equipment, in dense, highly populated areas, exposing not only workers but also residents in the surrounding communities.

Our estimates highlight the need for remediation of these sites, with a focus on addressing the key pollutant and dominant environmental medium. High-dose, mass poisonings periodically come to worldwide attention, such as recent events in Nigeria and Senegal, prompting immediate focus and remediation (Dooyema et al. 2012; Haefliger et al. 2009). However, exposures from most toxic waste sites continue unabated. Research has documented that waste site remediation can be cost-effective while reducing toxic exposures (Guerriero et al. 2011; Jones et al. 2011).

We must note several limitations of this analysis. We examined only eight chemicals and restricted the analysis to only one chemical per site. Persons living near toxic waste sites are often exposed to multiple chemicals simultaneously (DeRosa et al. 1996; Hu et al. 2007; Vrijheid 2000). Therefore, health effects may be increased or decreased due to the existence of coexposures and the potential for synergistic or antagonistic effects. For example, Claus Henn et al. (2011) documented a synergistic effect between lead and manganese in a Mexico City pregnancy cohort, with the impact of lead on child neurodevelopment increasing in the group with higher levels of manganese.

For most of the chemicals, we assigned only one cancer and one noncancer health effect. In addition, only a limited number of diseases have an associated DW, which prevented the inclusion of some health effects. For example, exposure to hexavalent chromium can cause nasal perforation. However, there is no DW for nasal perforation, so this health effect was not included in the analysis. In several cases there were no specific DWs that aligned properly with the projected health effect. Because there is no DW for liver toxicity, for example, we applied the DW for advanced hepatic disease to those chemicals known to cause liver toxicity. Although the major health effect of mercury is the impact of *in utero* methylmercury exposure on neurodevelopment, we were unable to capture this health effect for various reasons (e.g., limited methylmercury samples, no methylmercury biomonitoring). An additional source of uncertainty is the calculation of YLD for cancer, in which each cancer stage was assigned a different duration and DW.

Limited environmental sampling occurred at most sites, forcing us to extrapolate results of several samples to the entire population at risk. Biomarkers of exposure were not obtained, so we were unable to confirm completed pathways of exposure. In the case of lead, we attempted to offset this limitation by utilizing the U.S. EPA’s IEUBK model and ALM, which predict BLLs expected as a consequence of environmental lead exposure. However, these models may overestimate BLLs when predicted BLLs are > 30 µg/dL given the uncertainty in the relationship between environmental lead levels and BLLs at this level (Hogan et al. 1998). It is also likely that the actual exposures to the pollutants vary, with some individuals being exposed to lower levels. Despite evidence of prenatal exposure to environmental toxicants causing adverse health effects (Wigle et al. 2008), our analysis did not account for effects of prenatal exposures other than lead.

In addition, we assumed that exposures continued for a lifetime because there are no established waste site remediation programs in most LMICs. Although complete elimination of the toxic exposure may not be feasible for each site, a reduction in high-level exposure would decrease our disease burden estimates. Remediation of all sites such that pollutant concentrations are below international standards could save 798,405 DALYs_(3,1)_. Finally, a key limitation of this analysis is its reliance on slope factors, reference doses, and reference concentrations, largely based on animal testing. These regulatory values may overestimate the disease burden given the limitations of animal testing and the assumptions required to extrapolate toxicity data from animals to humans (e.g., applying uncertainty factors). Acknowledging these limitations, we believe our analysis presents the best possible estimate of the burden of disease from these sites given current data.

Further research should better define the specific exposures occurring at toxic waste sites in LMICs by linking environmental sampling levels, biomarkers of disease, and health outcomes and focusing on uniquely vulnerable populations such as women who are pregnant, children, and the elderly. Such enhanced surveillance data will help provide context when comparing toxic waste sites with more recognized public health threats. This research should not preclude the immediate remediation of existing sites given the disease and resulting costs to society that result from such exposures. Given that the majority of the DALYs estimated for the eight chemicals evaluated were due to lead and chromium exposures, remediation could be facilitated by selectively targeting lead- and chromium-contaminated sites.

## Conclusions

This study documents that chemical pollutants from toxic waste sites are a large and heretofore insufficiently studied public health problem in the three low- and middle-income Asian countries that we examined (India, Indonesia, and the Philippines). Disease and death caused by toxic chemicals contribute to the total burden of disease in these countries. We estimate that > 8 million persons in these countries suffered disease, disability, or death from exposures to industrial contaminants in 2010, resulting in 828,722 DALYs_(3,1)_. These findings underscore the urgent need for toxic waste sites around the world to be characterized and remediated and for the health of affected populations to be monitored.
